# MS-UNet: A Hybrid Network with a Multi-Scale Vision Transformer and Attention Learning Confusion Regions for Soybean Rust Fungus

**DOI:** 10.3390/s25175582

**Published:** 2025-09-07

**Authors:** Tian Liu, Liangzheng Sun, Qiulong Wu, Qingquan Zou, Peng Su, Pengwei Xie

**Affiliations:** 1 School of Electromechanical Engineering, Beijing Information Science and Technology University, Beijing 100192, China; liutian@bistu.edu.cn (T.L.); 20192260@bistu.edu.cn (Q.W.); 2023020109@bistu.edu.cn (Q.Z.); 2 School of Optoelectronics, Beijing Information Science and Technology University, Beijing 100192, China; 2023030031@bistu.edu.cn; 3 School of Artificial Intelligence, Beijing Normal University, Beijing 100875, China

**Keywords:** deep learning, image processing, *Phakopsora pachyrhizi*, U-Net, feature extraction

## Abstract

Soybean rust, caused by the fungus *Phakopsora pachyrhizi*, is recognized as the most devastating disease affecting soybean crops worldwide. In practical applications, performing accurate *Phakopsora pachyrhizi* segmentation (PPS) is essential for elucidating the morphodynamics of soybean rust, thereby facilitating effective prevention strategies and advancing research on related soybean diseases. Despite its importance, studies focusing on PPS-related datasets and the automatic segmentation of *Phakopsora pachyrhizi* remain limited. To address this gap, we propose an efficient semantic segmentation model named MS-UNet (Multi-Scale Confusion UNet Network). In the hierarchical Vision Transformer (ViT) module, the feature maps are down-sampled to reduce the lengths of the keys (K) and values (V), thereby minimizing the computational complexity. This design not only lowers the resource demands of the transformer but also enables the network to effectively capture multi-scale and high-resolution features. Additionally, depthwise separable convolutions are employed to compensate for positional information, which alleviates the difficulty the ViT faces in learning robust positional encodings, especially for small datasets. Furthermore, MS-UNet dynamically generates labels for both hard-to-segment and easy-to-segment regions, compelling the network to concentrate on more challenging locations and improving its overall segmentation capability. Compared to the existing state-of-the-art methods, our approach achieves a superior performance in PPS tasks.

## 1. Introduction

Asian soybean rust (ASR) holds great significance in the prevention of and research into related soybean diseases [1]. Since the 20th century, ASR has inflicted substantial losses on global soybean production, causing annual losses ranging from 10% to 30% and resulting in tens of billions of dollars of economic losses [2]. In soybean disease prevention and research, PPS is an essential step in understanding the morphodynamics of ASR and providing a comprehensive analysis of soybean growth conditions [3]. However, manual segmentation of *Phakopsora pachyrhizi* is laborious and inefficient and necessitates prior knowledge training for labelers in relevant fields. Given the scarcity of research on PPS-related datasets or automatic PPS, automated PPS holds significant practical application potential.

Numerous methods have been proposed for semantic segmentation of images, broadly categorized into traditional and deep-learning-based methods. Traditional methods typically employ classical image processing approaches that extract primary features using an object’s color, shape, or spatial position relationships. These methods generally offer faster inference speeds but are heavily dependent on manually designed features, exhibit a poor generalization performance for objects with varying characteristics, and are prone to pixel misclassification and sensitivity to variations in illumination in images. Recently, deep-learning-based methods have gained prominence as a promising solution to image semantic segmentation problems due to their robust nonlinear expression capabilities. For instance, the fully convolutional network (FCN) [4]-based method remains a popular framework for its ease of implementation and exceptional feature extraction capabilities. Additionally, a ViT adapts pure transformers to image classification tasks, opening the door to self-attention applications in other computer vision tasks. Numerous follow-up models have been developed, yielding an improved performance on many natural image datasets, such as PASCAL VOC and Cityscapes.

Nonetheless, applying deep-learning-based methods to ASR is not straightforward and faces challenges, such as blurred target boundaries and a heavy network architecture. Although state-of-the-art methods can be used to complete ASR, images of *Phakopsora pachyrhizi* differ from natural images and are observed through high-throughput microscopy. Conventional semantic segmentation networks typically employ an encoder–decoder structure combined with max-pooling and upsampling operations, which inevitably results in the loss of spatial information and a suboptimal performance in dense prediction tasks. As images of *Phakopsora pachyrhizi* lack the sharp borders characteristic of natural images, pooling layers further contribute to edge information loss and inaccurate segmentation results. Researchers often address this issue by maintaining high-resolution feature maps [5] or reducing the number of pooling layers during the encoding process [6]. However, these methods significantly increase the computational and parameter requirements, hindering the practical deployment of the model. Furthermore, although ViTs [7] have demonstrated a strong performance in computer vision, the self-attention mechanism’s $O(n^{2})$ time and space complexity for sequence length results in substantial training and inference overheads. Natural images are typically trained and inferred using ordinary-sized images (e.g., 224×224), which is computationally acceptable. In contrast, high-throughput microscopy images of *Phakopsora pachyrhizi* are generally small, sparse, and large. The $O(n^{2})$ time and space complexity further diminishes the advantages of deep-learning-based methods over image-based methods. A more suitable approach should involve an efficient model designed for *Phakopsora pachyrhizi*’s features, rather than the direct adoption of state-of-the-art ViT models.

To address these limitations, we propose a novel encoder–decoder-based model called the MS-UNet network, which takes into account the efficiency, accuracy, and robustness of PPS. The architecture of the proposed network is based on a hybrid transformer structure and is trained end to end, comprising the following designs: (1) We introduce a progressive hierarchical ViT encoder to make our network flexible for learning multi-scale and high-resolution features, reducing the resource consumption of the transformer and emulating the convolutions’ inductive bias as the network deepens. Specifically, our network has a hierarchical structure like a CNN, and we decrease the sequence length of the keys and values when computing the patch interactions for self-attention, significantly reducing the overhead with minimal impact on performance. Additionally, since positional encodings trained on small datasets are often insufficiently robust, we employ depthwise separable convolution to incorporate position information after each self-attention block. (2) To mitigate the loss of spatial information caused by pooling layers, we propose a multi-branch decoder that uses the network output to identify low-confidence pixels and forces additional branches to learn hard-to-segment locations. The proposed network achieves a competitive performance on PPS tasks, and our contributions are summarized as follows:

- We design a lightweight progressive hierarchical ViT encoder capable of learning multi-scale and high-resolution features;
- We develop a multi-branch decoder that leverages low-confidence pixels in the output to compel the network to focus on challenging regions that are difficult to segment;
- The proposed MS-UNet is tailored to the unique characteristics of soybean rust imagery and achieves satisfactory results on the PPS task.

## 2. Related Work

### 2.1. Semantic Segmentation

Semantic segmentation extends image classification from the image level to the pixel level [8,9,10,11]. In early deep learning applications, semantic segmentation was regarded as a pixel-wise classification task, unable to accomplish structured predictions [12,13]. Subsequently, Long et al. [4] proposed an FCN to address the structured prediction problem by integrating encoder–decoder architectures, becoming the predominant approach to semantic segmentation. Researchers then focused on enhancing the FCN from various perspectives [5,6,14,15,16,17,18,19]. To alleviate the spatial information loss caused by pooling layers, researchers typically maintained high-resolution feature maps [5,16] or reduced the number of pooling layers during the encoding process [6,18,19]. Chen et al. [6] proposed a method employing atrous convolution (dilated convolution) to expand the receptive field while avoiding down-sampling operations. Deeplabv3+ [19] introduced a simple and effective encoder–decoder FCN architecture by combining atrous spatial pyramid pooling. Sun et al. [16] consistently maintained a high-resolution branch to prevent spatial information loss and obtained various receptive fields through dense connections between different scales. However, these methods considerably increased the computational load and the number of parameters, which is unfavorable for actual model deployment. In contrast, our approach adds only two additional decoder branches, providing a clear advantage in terms of the computational overhead.

### 2.2. Vision Transformers

Transformers were initially proposed by [7] for translation tasks and quickly gained popularity across various NLP tasks. These models rely on self-attention mechanisms to capture long-range dependencies among tokens (words) in a sentence. The success of transformers in NLP has inspired several methods for computer vision tasks [20,21,22,23,24,25,26,27]. Ref. [20] demonstrated that self-attention is an instantiation of non-local means and used it to achieve gains in video classification and object detection. Ref. [21] developed a simple local self-attention layer suitable for both small and large inputs, outperforming the convolutional baseline for both image classification and object detection. Ref. [22] proposed a generic formulation for capturing long-range feature interdependencies via universal gathering and distribution functions. Ref. [24] proposed FBoT-Net, a focal bottleneck transformer network that effectively detects small green apples in complex orchard environments. As proposed in [25], MobileViTFace is a lightweight sheep face recognition model that combines convolutional and transformer structures, specifically using the cutting-edge ViT approach. It achieved a 97.13% recognition accuracy on 7434 sheep face images, outperforming lightweight convolutional models, while reducing the parameters and FLOPs compared to these values for ResNet-50 and providing real-time recognition results on edge devices like the Jetson Nano.

Although ViTs have achieved an exceptional performance in computer vision, the self-attention mechanism has an $O(n^{2})$ time and space complexity for sequence length, resulting in significant overheads during training and inference. To address this, we optimize the transformer for the characteristics of images of *Phakopsora pachyrhizi* and overcome numerous challenges when adapting self-attention to PPS tasks.

## 3. The Method

The U-Net network has demonstrated its ability to use a limited amount of data for model training in order to achieve pixel-level image prediction. Furthermore, the U-Net network has been shown to achieve a high level of segmentation performance in image processing [28,29,30,31,32,33,34,35,36,37,38]. In this work, the U-Net network serves as the foundation for constructing a segmentation network tailored to PPS tasks.

**Figure 1:**
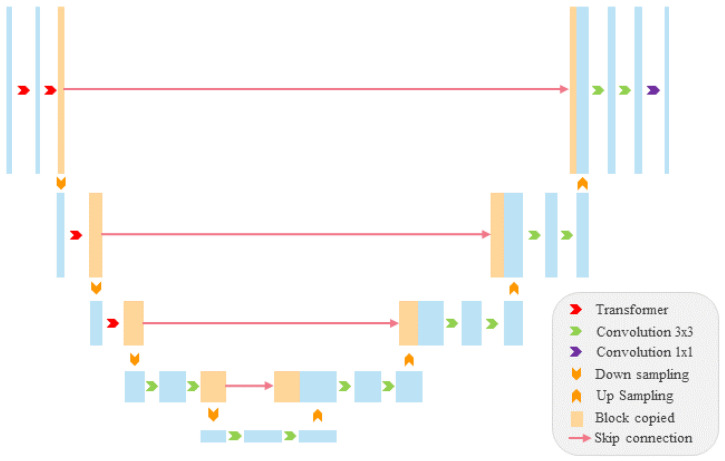
MS-UNetnetwork structure.

We designed a hybrid network structure, as depicted in Figure 1, and named it MS-UNet to incorporate the self-attention mechanism into PPS tasks. The segmentation network employs a U-Net architecture, where a transformer is utilized for the encoder part and three convolution-based decoders are employed for the decoder part. Figure 1 illustrates the network structure of a single decoder.

This study presents the development of a multi-branch segmentation network for high-precision segmentation of images of *Phakopsora pachyrhizi*. The objective of the network is to accurately segment regions in images that include both difficult and easy-to-segment areas. The network comprises a shared encoder and two distinct segmentation network branches. When an image is input into the multi-branch segmentation network, the encoder first extracts its features. These features correspond to both difficult and easy segmentation regions. The extracted features are then passed to three independent decoders, each tailored to these image characteristics. During the training of the complete multi-branch segmentation network, labeled *Phakopsora pachyrhizi* images are used as training data. Consequently, the most crucial aspect of the multi-branch segmentation network lies in devising suitable encoders and decoders for the network.

### 3.1. The Progressive Hierarchical VIT Encoder

**Figure 2:**
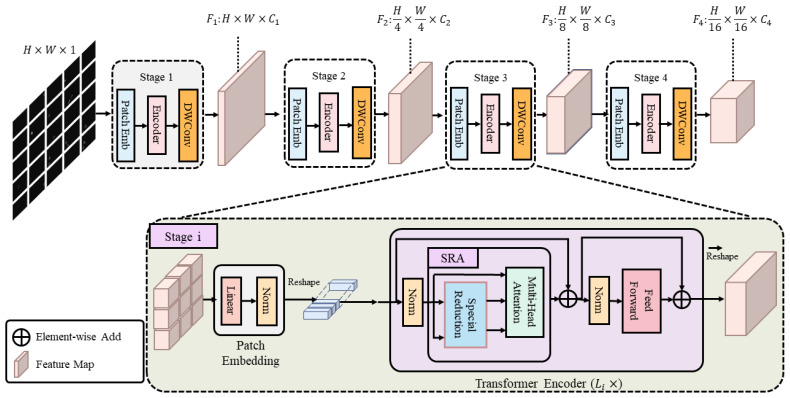
In the encoder section, the entire model is divided into four stages, each consisting of a patch embedding layer and a Li-layer transformer encoder. The output resolution of these four stages progressively decreases from high (4-stride) to low (32-stride) in a pyramidal fashion. Each execution of the transformer encoder corresponds to one level of down-sampling.

The Pyramid Vision Transformer (PVT) [39] introduces a pyramid that gradually shrinks and an attention layer that reduces the spatial dimensions to obtain high-resolution and multi-scale feature maps while minimizing the consumption of memory and computing resources. Based on the PVT, we develop a new encoder architecture, which can be seen in Figure 2.

Figure 2 depicts the structure of the encoder. A patch embedding layer and transformer encoder layers make up the architecture shared by all tiers. In the PVT structure, we add depth-separable convolution instead of the original position encoding. This approach has been demonstrated in [40].

Given an input image H×W×3 in size, we first partition it into $\frac{HW}{4^{2}}$ patches 4×4×3 in size. The flattened patches are then passed through a linear projection to produce embedded patches with a size of $\frac{HW}{4^{2}}\times C_{1}$. Following this, a transformer encoder with $L_{1}$ layers is employed to encode the embedded patches, and the output is reshaped into a feature map $F_{1}$ with the dimensions $\frac{H}{4}\times \frac{W}{4}\times C_{1}$. Similarly, by using the feature map from the previous step as the input, we obtain the subsequent feature maps $F_{2}$, $F_{3}$, and $F_{4}$, which have strides of 8, 16, and 32 pixels relative to the input image, respectively. The feature pyramid of $F_{1}$, $F_{2}$, $F_{3}$, and $F_{4}$ enables us to learn multi-scale and high-resolution features.

The PVT reduces the memory overhead through a specialized reduction, which is based on the principle of decreasing the sequence length of K and V. We achieve this using a novel approach. The multi-head self-attention (MHSA) module [7], on which the transformer is based, allows the model to jointly infer attention from multiple representation subspaces. The concatenated results from different heads are then transformed using a feed-forward network. In this study, we use eight heads, and for the sake of clarity in the subsequent formulation and illustration, the multi-head dimensions are omitted here for simplicity. Consider the input feature map $X\in R^{C\times H\times W}$, where *H*, *W*, and *C* represent the height, width, and number of channels of the feature map, respectively.

To project *X* onto the query, key, and value embeddings, *Q*, *K*, and *V* $\in R^{d\times H\times W}$, we employ three 1×1 convolutions, where *d* is the dimension of each head’s embedding. After being flattened, *Q*, *K*, and *V* are transposed into sequences of size n×d, where n=HW. Self-attention is computed through a scaled dot-product, as shown in Equation (1).

(1) $$\mathrm{Attention}\left(Q,K,V\right)=\underset{U}{\underset{︸}{\mathrm{softmax}\left(\frac{QK^{⊤}}{\sqrt{d}}\right)}}V$$

*U* can also be referred to as the similarity matrix or the context aggregating matrix.

More precisely, $U_{i}=$$\mathrm{softmax}\left(\frac{QiK^{⊤}}{\sqrt{d}}\right),U_{i}\in R^{1\times n}$, and $U\in R^{n\times n}$. The context aggregating matrix for the *i*-th query computes the normalized pairwise dot-product between $U_{i}$ and each element in the keys. The weights used to extract context information from the data are then derived from the context aggregating matrix. In this way, self-attention is effective at capturing long-range dependencies and inherently possesses a global receptive field. Additionally, the context aggregating matrix is sensitive to input data for enhanced feature aggregation. However, the dot-product of n×d matrices results in $O\left(n^{2}d\right)$ complexity. When the resolution of a feature map is high, *n* is often significantly larger than *d*; consequently, the sequence length dominates the self-attention computation, making self-attention infeasible for high-resolution feature maps. For instance, n= 50,176 for 224×224 feature maps.

We reduce the computational effort by shortening the length of the *K* and *V* sequences. The main idea is to project the key and value using two projections, $K,V\in R^{n\times d}$, into low-dimensional embeddings $K_{1},V_{1}\in R^{k\times d}$, where k=hw≪n, and *h* and *w* are the scaled-down dimensions of the feature map after sub-sampling. Now, the self-attention is shown in Equation (2).

(2) $$\mathrm{Attention}\left(Q,K_{1},V_{1}\right)=\underset{\bar{U}:n\times k}{\underset{︸}{\mathrm{softmax}\left(\frac{QK_{1}^{⊤}}{\sqrt{d}}\right)}}\underset{k\times d}{\underset{︸}{V_{1}}}$$

This reduction in computational complexity brings it down to O(nkd). It is worth noting that any down-sampling operation, including average/max-pooling or stridden convolutions, can be utilized as the projection to low-dimensional embedding. In our method, the feature map is down-sampled using convolution and then reduced to a size of 4 using bilinear interpolation. The original expressions for Q,K,V are $Q=W^{Q}X$, $K=W^{K}X$, and $V=W^{V}X$, where $W^{Q},W^{K},W^{V}$ are the corresponding feature matrices. We modify the expressions of the K,V sequence length as follows: $Q=W^{Q}X$, $K=W^{K}\mathrm{Conv}\left(X\right)$, and $V=W^{V}\mathrm{Conv}\left(X\right)$.

Furthermore, we replace the original position encoding of the PVT with a depth-separable convolution. The purpose of positional encoding is to address the alignment invariance in the self-attention operation, and one approach is to break the invariance in subsequent self-attention operations by introducing positional information at the input [7]. Another approach is to delve deep into the network and introduce some information at the self-attention operation, thereby breaking this invariance [40]. The method employed here follows the second approach, which breaks the invariance by reshaping the tokens into feature maps and then aggregating tokens that are not adjacent to each other through depth-separable convolution. Consequently, when the tokens are rearranged, the convolutionally aggregated tokens will change, thus breaking the invariance.

### 3.2. The Multi-Branch Decoder

Inspired by [41], which manually marks thick and thin vessels into two different labels and then trains the two different labels using two U-Net network decoders, the final output results are fused and used as the input for another U-Net network. This network is then trained with the original labels to obtain the final results. However, the downside of this approach is the need to manually segment the hard-to-segment regions, which is very time-consuming and labor-intensive. High-throughput images of *Phakopsora pachyrhizi* differ from general images, as they exhibit fuzzy boundaries and belong to the hard-to-segment category. Consequently, we design a network architecture that automatically distinguishes hard-to-segment regions through coarse segmentation to obtain low-confidence regions. Then, images with coarse segmentation, hard-to-segment regions, and easy-to-segment regions are trained by three decoders, ultimately serving as the primary source of information for the fusion network.

**Figure 3:**
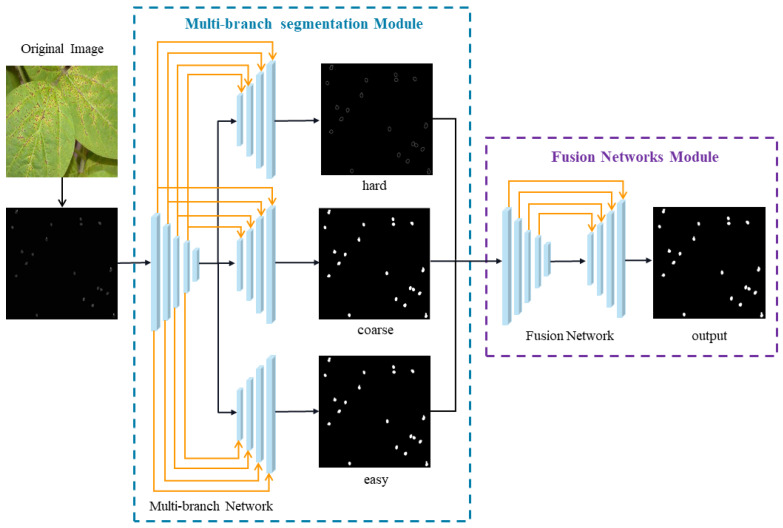
The proposed multi-branch decoder.

This multi-branch segmentation model comprises a universal encoder and several distinct network branches with different loss functions, which can handle different tasks effectively and provide an efficient method for dealing with *Phakopsora pachyrhizi* image segmentation. Based on the results outlined above, we propose a new image hybrid depth segmentation network in this paper to enhance the segmentation accuracy for *Phakopsora pachyrhizi* images. The overall structure of the multi-branch segmentation approach is depicted in Figure 3. The fundamental structure of the approach consists of two distinct parts: the multi-branch segmentation network and the fusion network. The multi-branch network employs an MS-UNet structure, with a hierarchical ViT model for the encoder part and a convolutional network for the decoder part. For the fusion network, U-Net is used.

The multi-branch segmentation network automatically distinguishes the hard- and easy-to-segment regions of images of *Phakopsora pachyrhizi* and feeds them into the multi-branch segmentation network for training. Segmentation images from the respective network branches are obtained and used as input for the fusion network, along with the coarse segmentation images. Due to the structural similarity between the hard and easy segmentation regions, there is an overlap between these two coarse segmentation images. Therefore, a fusion network is employed to merge these two images with coarse-grained segmentation. The coarsely segmented images generated by the multi-branch segmentation network are used to train the fusion network, which is then given the original ground-truth values from the *Phakopsora pachyrhizi* dataset. This process is iteratively performed. The image is binarized and subsequently processed to produce the final segmentation result of *Phakopsora pachyrhizi* using the fusion result. This technique has the potential to effectively remove the overlap between the segmented images of these two distinct regions, thereby enhancing the overall segmentation accuracy.

**Figure 4:**
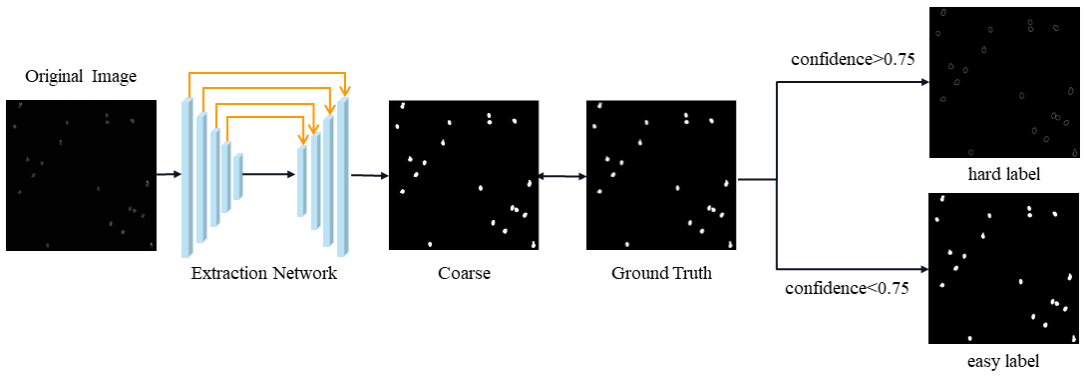
The label extraction network.

Our proposed decoder network aims to automatically acquire hard-to-segment regions and compel the network to learn. Therefore, we design a network architecture that automatically extracts hard-to-segment and easy-to-distinguish labels, as illustrated in Figure 4. First, the input information on the images is acquired through the designed hierarchical ViT encoder structure, and rough segmentation results are obtained. Next, the rough segmentation result is compared with the ground truth, and pixels with a confidence greater than 0.75 are considered well-segmented areas, while pixels with a confidence of less than 0.75 are considered poorly segmented areas. We set two masks both with 1 s, $Aux_{-}easy$ and $Aux_{-}hard$, and dimensions of C×H×W. The formula for generating the label is as follows:

(3) $$Aux_{-}easy_{\left(x,y\right)}=\left\{\begin{array}{cc} 1, & \;\mathrm{if}\;\delta (x,y)>0.75\\ 0, & \;\mathrm{if}\;\delta (x,y)<0.75 \end{array}\right.$$

(4) $$Aux_{-}hard_{\left(x,y\right)}=\left\{\begin{array}{cc} 0, & \;\mathrm{if}\;\delta (x,y)<0.75\\ 1, & \;\mathrm{if}\;\delta (x,y)>0.75 \end{array}\right.$$

δ(x,y) denotes the confidence level of each pixel point in the rough segmentation results.

(5) $$Hard_{-}label=GT\cdot Aux_{-}\mathrm{hard}\left(x,y\right)$$

(6) $$Easy_{-}label=GT\cdot Aux_{-}easy\left(x,y\right)$$

GT indicates the ground truth.

These two masks, $Aux_{-}easy$ and $Aux_{-}hard$, will be used to identify easy-to-segment and hard-to-segment regions, respectively. By applying these masks, the decoder network will focus on learning the segmentation patterns for each type of region separately, leading to an improved segmentation performance overall.

To obtain the labels for the problematic region, we set the values with a confidence of less than 0.75 in the mask to 1 and values greater than 0.75 to 0. We then multiply this mask with the ground truth. On the other hand, to obtain the labels for the simple region, we set the values with a confidence greater than 0.75 in the mask to 1 and values less than 0.75 to 0. We then multiply this mask with the ground truth. In essence, the hard-to-segment region is masked to retain the region with low confidence, and the easy-to-segment region is masked to retain the region with high confidence. We then multiply these masks with the ground truth for *Phakopsora pachyrhizi* to obtain two types of labels.

### 3.3. The Loss Function

The proposed training process consists of coarse segmentation, the separation of hard/easy regions based on a confidence threshold, and fusion of the segmentation results. For a binary *Phakopsora pachyrhizi* segmentation (PPS) task, we employ the Binary Cross-Entropy (BCE) loss:

(7) $$L_{BCE}=-\frac{1}{n}\sum \left[y_{1}\log{\hat{y}}_{1}+\left(1-y_{1}\right)\log\left(1-{\hat{y}}_{1}\right)\right]$$

where $y_{1}$ is the ground-truth label probability, and ${\hat{y}}_{1}$ is the predicted probability.

The total loss is a weighted sum of the BCE losses from five branches: coarse segmentation, hard regions, easy regions, hard/easy fusion, and the final fusion network:

(8) $$Loss_{total}=\sum_{i=1}^{5}\alpha_{i}\;L_{BCE}\left({\mathrm{Pre}}_{i}\right)$$

${\mathrm{Pre}}_{i}$ is the output of branch *i*, and $\alpha_{i}$ is its weight coefficient.

## 4. Experiments

### 4.1. Databases

We present a curated dataset comprising 4414 original microscopy images and their corresponding manually annotated labels, documenting the morphological development of fungal spore buds. The original images were obtained from the publicly accessible Cell Image Library (CIL, National Center for Microscopy and Imaging Research, La Jolla, CA, USA; available at https://cellimagelibrary.org, accessed on 4 September 2025) and systematically organized into a time-series collection that captured the morphological changes and growth directions of spore buds under DMSO treatment conditions. The dataset spans the entire developmental timeline, covering approximately 90 to 210 min post-germination, with images acquired at fixed 15 min intervals. This ensures that the dataset includes a complete morphological evolution sequence, from the early germination stages to the late developmental phases. For stricter temporal generalization, we grouped the dataset by 9 exclusive time intervals. We then applied cross-validation: in each round, 7 intervals were used for training, with 1 for validation and 1 for testing (an 8:1:1 ratio). This procedure was repeated across all combinations. The dataset supporting this study has been deposited in Zenodo and is publicly accessible at https://doi.org/10.5281/zenodo.17004172, accessed on 4 September 2025.

### 4.2. Evaluation Metrics

We evaluate segmentation performance using four standard metrics: precision (Pre), recall (Re), Mean Intersection over the Union (MIoU), and F1-score (F1). Let $T_{P}$, $F_{P}$, $T_{N}$, and $F_{N}$ denote true positives, false positives, true negatives, and false negatives, respectively. For binary segmentation (K=1), the metrics are defined as

(9) $$Pre=\frac{T_{P}}{T_{P}+F_{P}}$$

(10) $$Re=\frac{T_{P}}{T_{P}+F_{N}}$$

(11) $$MIoU=\frac{1}{K+1}\sum_{i=0}^{K}\frac{\mathrm{TP}}{\mathrm{FN}+\mathrm{FP}+\mathrm{TP}}$$

(12) $$F1=\frac{2\times Pre\times Re}{Pre+Re}$$

These metrics jointly measure classification accuracy (Pre, Re), spatial overlap (MIoU), and the harmonic balance between precision and recall (*F1*).

We further adopt the Boundary Intersection over the Union (Boundary IoU) to evaluate the segmentation quality along object contours. Unlike the region-based IoU, which considers the overlap of entire regions, the Boundary IoU measures the consistency between the predicted and ground-truth boundary regions, making it more sensitive to boundary errors in images with fuzzy object contours such as fungal microscopy images. Formally, let *P* and *G* denote the predicted and ground-truth masks, respectively. Their boundary regions, ∂P and ∂G, are obtained by dilating the contours within a fixed distance. The metric is defined as:

(13) $$BoundaryIoU=\frac{\left|\partial P\cap \partial G\right|}{\left|\partial P\cup \partial G\right|},$$

where |·| denotes the number of pixels in a set, and ∩ and ∪ represent the intersection and union operations, respectively.

We also introduce the Shape Complexity Similarity (SCS) metric, which measures the similarity of the shape complexity between the predicted and ground-truth regions. Unlike traditional overlap-based metrics, SCS can quantify morphological similarity even when the boundaries are fuzzy, thereby providing a more robust assessment of structural preservation. In fungal image analysis, this metric can capture morphological variations across different strains or growth stages, complementing conventional metrics. The shape complexity of a region *R* is defined as

(14) $$C\left(R\right)=\frac{\mathrm{Perimeter}{\left(R\right)}^{2}}{4\pi \times \mathrm{Area}(R)}.$$

Then, SCS is computed as

(15) $$SCS=1-\frac{\left|C(P)-C(G)\right|}{\max\{C(P),C(G)\}},$$

where Perimeter(R) and Area(R) denote the perimeter length and area of region *R*, respectively. A higher SCS indicates better agreement in morphological complexity between the prediction and the ground truth.

### 4.3. Implementation Details

The training consists of two stages: multi-branch segmentation network training and fusion network training. In the multi-branch network, three ground truths are used: original *Phakopsora pachyrhizi* labels, automatically extracted hard regions, and easily distinguishable regions. BCE loss and the RMSprop optimizer (initial learning rate $1\times 10^{-5}$) are employed, with 300 training epochs. The fusion network is trained using the segmentation results from the multi-branch network and the original labels, following the same loss and optimization settings. All experiments are conducted on an NVIDIA GeForce RTX 4090 (24 GB) GPU, NVIDIA, Santa Clara, CA, USA).

## 5. Results and Discussion

### 5.1. Experiments with Automatic Label Generation

**Figure 5:**
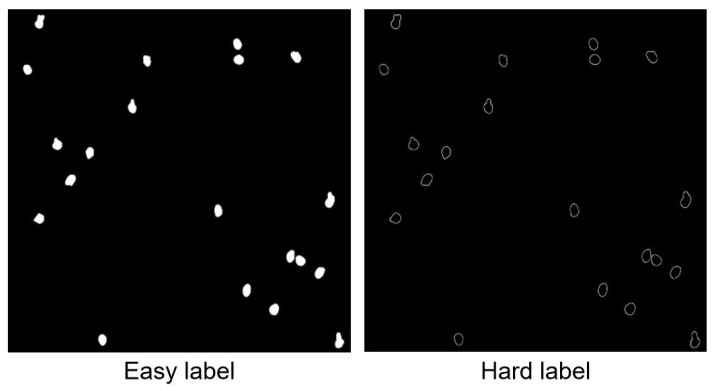
The two generated labels.

We first train a single-decoder MS-UNet to obtain coarse segmentation. The coarse results are then compared with the ground truth to identify hard-to-segment and easy-to-segment regions. This process enables the multi-branch segmentation network to focus on learning the hard-to-segment regions. As shown in Figure 5, difficult segmentation regions exist at the edge of the soybean rust fungus, which is why the multi-branch segmentation network is proposed to learn these challenging areas. The automatic extraction of labels and focusing on learning the boundary for soybean rust fungus can improve the segmentation accuracy and reduce spatial information loss.

Indeed, Figure 5 highlights the presence of difficult segmentation regions at the edges of the soybean rust fungus. This observation is the main reason behind proposing a multi-branch segmentation network to learn these challenging areas. In high-throughput microscopy images of soybean rust fungus, the boundaries are not as clear as those in natural images, making segmentation a complex task.

By automatically extracting labels and focusing on learning the boundaries of soybean rust fungus, the segmentation accuracy can be improved, and the loss of spatial information can be reduced. This approach helps the multi-branch segmentation network to effectively identify and segment hard-to-segment regions, resulting in a better overall segmentation performance compared to that of other methods.

### 5.2. Comparison with Other Methods

**Figure 6:**
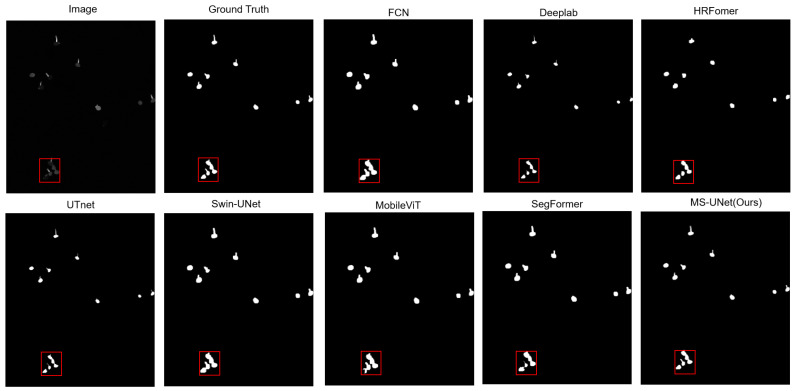
Segmentation results of different network structures on *Phakopsora pachyrhizi* dataset. Squares indicate the regions that are enlarged for detailed visualization.

**Figure 7:**
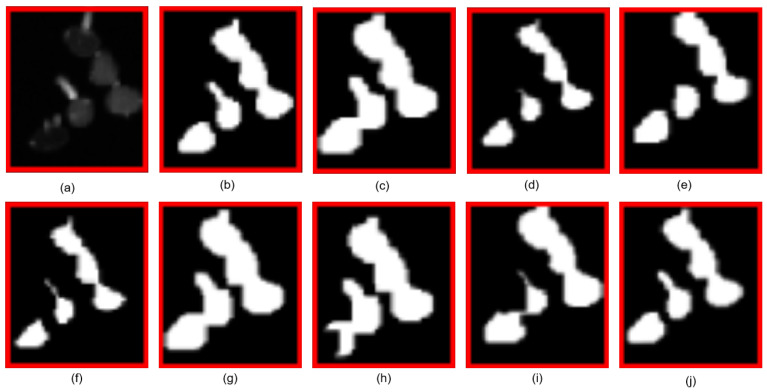
The details of a single *Phakopsora pachyrhizi* segmentation, where (**a**,**b**) denote the original image and the ground truth and (**c**–**j**) are the segmentation results of FCN, Deeplab, HRFormer, UTnet, Swin-UNet, MobileViT, SegFormer, and MS-UNet, respectively.

To demonstrate the details of the segmentation results better, an image of soybean rust fungus was used as the experimental subject. Experiments were conducted with traditional network structures like DeepLab and an FCN and advanced network structures such as UTNet, HRFormer, MobileVIT, Swin-UNet, and SegFormer. The experimental results are shown in Figure 6. As illustrated in Figure 6, in the soybean rust fungus (*Phakopsora pachyrhizi*) segmentation task, we observed significant differences in the performance of different algorithms. Figure 6 and Figure 7 (where Figure 7 shows the local magnification details) clearly demonstrate that the FCN, MobileVIT, and Swin-UNet exhibited overlapping phenomena when processing adjacent spores, leading to inaccurate segmentation results; while DeepLab, UTNet, HRFormer, and SegFormer avoided overlapping issues, they still had deficiencies in spore boundary localization and shape preservation, being prone to boundary blurring or shape distortion; our MS-UNet not only successfully avoided overlapping issues but also performed optimally in maintaining the complete spore morphology and boundary clarity.

To emphasize the model’s performance better, Table 1 presents the values of the evaluation metrics. The proposed MS-UNet achieves a superior performance across all evaluation metrics, including precision, recall, the mIoU, the F1-score, the Boundary IoU, and SCS, compared to those of the other methods. This demonstrates the effectiveness of the multi-branch segmentation network in learning and segmenting hard-to-segment regions in soybean rust fungal images.

The reason MS-UNet surpasses traditional networks such as the FCN, DeepLab, HRformer, and UTnet in segmenting soybean rust fungus is due to the addition of a hierarchical ViT structure to the encoder. This structure allows the network to learn multi-scale and high-resolution features. Furthermore, the model features a multi-task branching decoder, which forces the network to learn hard-to-segment regions, resulting in sharper edges in the segmented images.

### 5.3. An Ablation Study on Individual Components

In the ablation experiments (Table 2), the validity of the modules proposed in this paper is investigated. The model framework is based on U-Net, which is used as the benchmark. Table 2 demonstrates that adding the attention mechanism to the U-Net algorithm improves its performance, as seen with Ours-Multibranch. Each module designed in this paper positively impacts the algorithm’s improvement. Compared to those of the U-Net network, Pre, Re, MIoU, the F1-score, the Boundary IoU, andSCS are improved by 7.0%, 5.6%, 6.1%, 7.0%, 9.2%, and 7.0%, respectively. The best performance comes from the combined effect of the two modules, showing the effectiveness of the proposed approach.

The computational cost of each component is summarized in Table 3. To improve the overall performance of the model, each module introduces only a moderate increase in the computational overhead and inference latency, both of which remain within an acceptable range.

### 5.4. Ablation Experiments on the Threshold

The results of the threshold sensitivity analysis are presented in Table 4. Specifically, we evaluated multiple thresholds at 0.15 intervals, starting from 0.15. The experimental results indicate that a threshold of 0.75 yields the best overall performance, achieving a balanced improvement across key metrics such as the MIoU and F1-score. This finding validates the effectiveness of using 0.75 as the decision threshold to distinguish between easy and hard regions.

### 5.5. Computational Analysis

We consider the MS-UNet model to be highly suitable for practical deployment scenarios, particularly in medical image segmentation tasks with stringent real-time requirements. As shown in Table 5, MS-UNet contains only 19.21 M parameters, which is substantially fewer than those in classic models such as the FCN (134.3 M) and DeepLab (41.2 M). Its FLOPs are 31.2 G, also markedly lower than those of Swin-UNet (67.3 G) and HRFormer (89.2 G), enabling good portability in resource-constrained environments (e.g., on edge devices or mobile platforms). Moreover, the inference time on an RTX 4090 is only 27.6 ms for 512 × 512 inputs, significantly faster than HRFormer (52.7 ms) and Swin-UNet (39.8 ms), thereby satisfying real-time processing requirements such as 30+ frame per second video streams. In addition, the deployment potential of MS-UNet could be enhanced further through model compression techniques such as quantization or pruning.

## 6. Conclusions

To address the task of segmenting high-throughput microscopic images of *Phakopsora pachyrhizi* with blurred edges, a network model with a hierarchical ViT structure for the encoder and a multi-branch network for the decoder is proposed. An image fusion network is also designed for fusing images. The main contributions of this study are as follows:

- A segmentation dataset of *Phakopsora pachyrhizi* was produced manually for the related research.
- A multi-branch segmentation network was proposed to improve the segmentation accuracy in PPS by accurately segmenting difficult- and easy-to-segment regions from images of Phakopsora pachyrhizi.
- To ensure the segmentation performance of the multi-branch segmentation network, an upgraded U-Net network was proposed as the base segmentation network. Specifically, a combination of a transformer and convolution was used to create a progressive hierarchical ViT encoder capable of learning multi-scale and high-resolution features, resulting in a segmentation network suitable for the PPS problem.
- A fusion network was employed to solve the issue of overlapping regions in images of *Phakopsora pachyrhizi* by fusing two distinct sections of a multi-branch segmentation network.

Using the *Phakopsora pachyrhizi* dataset, the proposed method demonstrates a good segmentation performance for PPS tasks compared to that of other recent segmentation methods and traditional segmentation techniques. Notably, it excels in edge contour extraction for spore images. Further improvements in the segmentation accuracy are needed. Theoretically, this method is applicable to other segmentation tasks, particularly when there are challenging parts of the image to be segmented.

## Figures and Tables

**Table 1:** Segmentation results of different methods on *Phakopsora pachyrhizi* dataset. *Note*: Bold numbers indicate the best performance in each column.

Methods	Pre	Re	MIoU	F1	Boundary IoU	SCS
FCN [4]	0.965 ± 0.012	0.925 ± 0.018	0.945 ± 0.011	0.944 ± 0.013	0.885 ± 0.015	0.832 ± 0.014
DeepLab [6]	0.901 ± 0.019	0.932 ± 0.016	0.895 ± 0.017	0.888 ± 0.018	0.856 ± 0.021	0.857 ± 0.019
HRformer [5]	0.785 ± 0.023	0.928 ± 0.020	0.862 ± 0.022	0.851 ± 0.021	0.822 ± 0.025	0.848 ± 0.023
UTnet [42]	0.923 ± 0.014	0.985 ± 0.008	0.951 ± 0.010	0.953 ± 0.011	0.899 ± 0.013	0.908 ± 0.012
SegFormer [43]	0.922 ± 0.009	0.938 ± 0.012	0.921 ± 0.008	0.935 ± 0.010	0.877 ± 0.014	0.886 ± 0.011
Swin-UNet [44]	0.933 ± 0.011	0.958 ± 0.010	0.945 ± 0.009	0.946 ± 0.010	0.878 ± 0.012	0.894 ± 0.011
MobileViT [45]	0.906 ± 0.016	0.905 ± 0.015	0.913 ± 0.014	0.906 ± 0.015	0.851 ± 0.017	0.872 ± 0.016
MS-UNet	**0.987 ± 0.012 **	**0.959 ± 0.014 **	**0.963 ± 0.012 **	**0.967 ± 0.009 **	**0.913 ± 0.011 **	**0.935 ± 0.013 **

**Table 2:** Performance comparison of different model components. *Note*: Bold numbers indicate the best performance in each column. The symbol “×” indicates that the module is not included, while “✓” indicates that the module is included.

Configuration	Attention	Multi- Branch	U-Net Fusion	Pre	Re	MIoU	F1	Boundary IoU	SCS
U-Net [46]	×	×	×	0.917	0.903	0.902	0.897	0.821	0.865
Ours-Attention	✓	×	×	0.938	0.921	0.913	0.921	0.835	0.878
Ours-Multibranch	×	✓	×	0.951	0.925	0.936	0.941	0.851	0.892
Ours-Attention-Multibranch	✓	✓	×	0.967	0.927	0.945	0.947	0.870	0.907
Ours	✓	✓	✓	**0.987 **	**0.959 **	**0.963 **	**0.967 **	**0.913 **	**0.935 **

**Table 3:** Computational efficiency comparison of different model components. *Note*: The symbol “×” indicates that the module is not included, while “✓” indicates that the module is included.

Configuration	Attention	Multi- Branch	U-Net Fusion	Parameters (M)	FLOPs (G)	Maximum Memory Usage (MB)	Inference Time (ms)
U-Net [46]	×	×	×	7.76	6.2	642	12.4
Ours-Attention	✓	×	×	10.23	14.8	798	16.7
Ours-Multibranch	×	✓	×	15.67	18.6	1245	20.8
Ours-Attention-Multibranch	✓	✓	×	18.12	28.4	1487	25.3
Ours	✓	✓	✓	19.21	31.2	1576	27.6

**Table 4:** The performance of the model across different threshold values. *Note*: Bold numbers indicate the best performance in each column.

Threshold	*Pre*	*Re*	*MIoU*	*F1*	*Boundary IoU*	*SCS*
0.15	0.891	0.973	0.896	0.935	0.831	0.862
0.30	0.915	0.965	0.912	0.943	0.849	0.878
0.45	0.938	0.961	0.927	0.951	0.867	0.894
0.60	0.956	0.954	0.939	0.955	0.882	0.909
**0.75 **	**0.987**	**0.959 **	**0.963 **	**0.967 **	**0.913 **	**0.935 **
0.90	0.989	0.928	0.948	0.96	0.894	0.917

**Table 5:** Computational efficiency comparison of different methods.

Methods	Parameters (M)	FLOPs (G)	Maximum Memory Usage (MB)	Inference Time (ms)
SegFormer [43]	3.7	8.4	648	11.2
MobileViT [45]	6	4.9	512	9.6
UTnet [42]	9.53	15.8	782	16.4
Swin-UNet [44]	27	67.3	2184	39.8
HRformer [5]	37.9	89.2	3048	52.7
DeepLab [6]	41.2	52.7	1724	26.5
FCN [4]	134.3	148.6	2856	18.3
MS-UNet	19.21	31.2	1576	27.6

## Data Availability

The dataset supporting this study has been deposited in Zenodo and is publicly accessible at [https://doi.org/10.5281/zenodo.17004172, accessed on 4 September 2025].
